# Molecular phylogeny and pathogenicity of indigenous *Beauveria bassiana* against the tomato leafminer, *Tuta absoluta* Meyrick 1917 (Lepidoptera: Gelechiidae), in Ethiopia

**DOI:** 10.1186/s43141-021-00227-x

**Published:** 2021-08-26

**Authors:** Birhan Aynalem, Diriba Muleta, Juan Venegas, Fassil Assefa

**Affiliations:** 1 Institute of Biotechnology, Addis Ababa University, Addis Ababa, Ethiopia; 2 Cellular and Molecular Biology Program, Faculty of Medicine, Institute of Biomedical Sciences, University of Chile, Santiago, Chile; 3 Department of Microbial, Cellular and Molecular Biology, College of Natural and Computational Sciences, Addis Ababa University, Addis Ababa, Ethiopia

**Keywords:** Biocontrol, Chitinolytic enzyme, Entomopathogenic fungi, Mycoinsecticides

## Abstract

**Background:**

*Tuta absoluta* Meyrick 1917 (Lepidoptera: Gelechiidae) is an invasive, pesticide resistant, and a major treat of tomato production in the world. It needs effective management options that naturally infect the insect without causing any identified side effects. Entomopathogenic fungi (EPF) are the most important options. However, geographic origin and climatic condition apparently creates genetic variation among EPF strains that influence on their pathogenicity. Thus, screening of effective EPF strains from the local source is vital to develop environmental friendly pest control tactic for *T. absoluta*.

**Results:**

In this study, 27 indigenous *Beauveria* were isolated from the various types of soil and 12 of the isolates were screened based on their biological efficiency index (BEI). These isolates scored 65.7–95.7% and 68.3–95% of mortality against second and third instar larvae of *T. absoluta* at concentration of 1 × 10^7^spores·ml^-1^ in 7 days post inoculation, respectively. Out of these, five (18.5%) isolates scored above 90% mortality on both instar larvae with LT_50_ value of 3.33 to 5.33 days at the lowest (10^4^ spores·ml^-1^) and 1.93 to 3.17 days at highest (10^8^ spores·ml^-1^) spore concentrations and has LC_50_ value of 1.5 × 10^3^ to 1.1× 10^5^ spores·ml^-1^. Moreover, isolates exhibited the promising mortality better (1.5 × 10^6^ to 3.5 × 10^7^ spores·ml^-1^), sporulated over the larval cadavers, well grown at optimal temperature, and produced chitinolytic enzymes. Molecular analysis showed that isolates have nearly monophyletic characters and grouped under species of *Beauveria bassiana*.

**Conclusion:**

Different types of soil in Ethiopia are an important source of *B. bassiana*, and these isolates showed promising pathogenicity against *T. absoluta*, which is crucial for ecofriendly biopesticide development. Although isolates were nearly monophyletic in phylogenetic study, five of them were highly effective in the laboratory bioassays against *T. absoluta*; however, further field evaluation is required for mass production.

## Background

Insect pests are the major threats of vegetable production. The emergence of new invasive pests by global climate change is aggravating the problem by facilitating fast reproduction, easy adaptation, and rapid dissemination of insects [1]. Thus, invasive pests are becoming main challenges of food security against the continuously rising human population in the world.

*Tuta absoluta* Meyrick 1917 (Lepidoptera: Gelechiidae) is a native tomato (*Solanum lycopersicum* L.) leafminer pest in South America for more than 50 years [2]. Later on, it has become invasive with its notorious spread starting from Spain in 2006 [3], and is currently recognized as an economically important pest of solanaceous plants worldwide [4, 5]. It is estimated that *T. absoluta* has infested 41 out of 54 African countries [6]. In Ethiopia, the pest has been detected since 2012 [7] and is causing more than 78% loss of tomato production [8]. Although tomato is the main host for feeding and oviposition of *T. absoluta*, several cultivated and wild solanaceous plants are serving as alternative hosts [9]. *T. absoluta* can cause up to 100% of damage on the tomato production if it is not managed timely [10].

The control difficulty of the insect emanates from the enormous amount of eggs produced by the female that lays 250 up to 300 on the plant leaves, hatched to feeding stage larvae, which feed on leaf mesophyll, bore into fruits and stem, as well as expose plants for secondary infection [11]. The mean time controlling option of the insect is mainly dependent upon the application of synthetic insecticides as primary solution [12]. However, the insect is fast developing resistance to individual chemicals [13] that forces farmers to try a combination of several types of inappropriate chemicals to improve the control. Continuous application and flooding of huge doses of inappropriate chemicals on the cropland has been aggravating environmental pollution, food contamination, and human health problems [14]. Therefore, this multiple problem is calling for environmentally friendly pest management alternatives.

These days, the biocontrol method is given attention as a promising technology to control insect pests as part of the integrated pest management (IPM) strategy [15]. Entomopathogenic fungi (EPF) are the recognized part of mycoinsecticidal IPM agents against several insect pests [16]. The United States of Environmental Protection Agency (USEPA) classified genus *Beauveria* as a biopesticide [17]. This insect pathogen is isolated from the soil [18, 19], insect cadavers [17], as well as plant tissues [20], and checked for their pathogenicity against several agriculturally important pests [21]. Out of these, *Beauveria bassiana* is highly pathogenic to many insect pests through the mechanism of spore germination, cuticle penetration, and mycelial dissemination mode of action inside the body [22], and is formulated and available in the market as biopesticides for many years.

Although this fungal species is commercialized as mycoinsecticides for more than decades and used for arthropod pest management [23], locally isolated strains showed better performance than formulated products. For instance, Wang and Zhang [24] identified virulent *B. bassiana* with 93% mortality against *Frankliniella occidentalis* Pergande from the local environment elsewhere indicating that the virulent strain can still be isolated from given environmental conditions with better adaptation. The geographic distance of origin apparently creates genetic variation among strains of *B. bassiana* [25]. This could possibly infer that distinct strains of *B. bassiana* might be found in different ecological zones of Ethiopia, which is imperative to explore entomopathogenically effective ones from the various locations to use for vegetable production by smallholder farmers with affordable economic return. Therefore, the objective of this study was isolation, molecular identification, and pathogenicity evaluation of local strains of *B. bassiana* against second and third instar larvae of *T. absoluta*, to validate and recommend for future biocontrol tactics.

## Methods

### Sample collection

The cropland and grassland soils were collected from the central rift valley area of Ethiopia. This area is potential for tomato production using irrigation system and known for *T. absoluta* infestation. The forest soil was collected from “Menagesha National Forest” that covers an altitude between 2574 and 2948 m above sea level (masl). Soil samples were taken from the tomato farm and undisturbed places of grassland and forest; each soil-sampling site was replicated three times; soils were pooled together, composited, and collected into 2-kg–capacity ethanol-sterilized (70%) polyethylene bags. A total of 43 soil samples were collected and brought into Applied Microbiology Laboratory, Addis Ababa University (AAU) for further work. For rearing the insect pest, *T. absoluta–*infected tomato leaves and fruits harboring larvae and pupae of the inset were collected from the same tomato-grown rift valley area.

### Rearing of *Tuta absoluta*

The *T. absoluta–*infected tomato leaves and fruits harboring larvae and pupae collected from the central rift valley area were transferred into 5-week-old pot-grown “Awash” cultivar tomato plants, kept under zipped cages constructed from wooden poles and meshed cotton cloth. Pot-growing tomato plants supplemented in rearing cages once per 2 weeks to make insect egg-laying process is continuous and emerging larval feed. Infected tomato leaves in the rearing cages were periodically inspected for larval development until the suitable instar larvae were obtained and the third generation (F3) of *T. absoluta* was used for EPF pathogenicity bioassay.

### Isolation of *Beauveria* species

The great wax moth (*Galleria mellonella*) reared in Ambo Plant Protection Research Centre (APPRC) according to the methods of Meyling [26] was used for EPF baiting. *Beauveria* were isolated from the soil using the *G. mellonella* baiting method [26]. Briefly, the third instar larvae of great wax moth were shocked for 10 s in hot (65 °C) water bath to reduce their fast movement within the soil and transferred into 1 1/2 L capacity of screw-caped glass jars filled with 1 kg of moisturized soil. The jars were inoculated with ten heat-shocked great wax moth larvae incubated at 30 °C for 10 days under complete dark condition.

The death of larvae in the soil was inspected every 3 days, and the moisture content of the soil was adjusted by gentle moistening with sterile water each time following the death inspection. Cadavers of dead larvae were carefully removed from the soil, surface sterilized by using sodium hypochlorite (3%) followed by ethanol (70%) for 3 min each, rinsed five times with sterile water, placed on the sterile plastic plates lined with UV serialized and moistened tissue paper, and incubated at room temperature under dark condition until fungal mycelia and spores outgrow. Then the spore was scraped using inoculating wire loop, transferred onto potato dextrose agar (PDA) medium supplemented with 0.03 g·l^-1^ of chloramphenicol and incubated at 28 °C for 20 days. Subculturing of isolates onto fresh PDA medium was used for purification, and pure cultures were maintained on agar slants at 4 °C for further work.

### Morphological identification

Morphology of the isolated fungi was characterized by using methods of Rehner et al. [27]. The cultural characteristics such as colony size, mycelial color, colony reverse, and color of conidial mass were examined from the PDA culture plates. Spore morphology such as shape and size were inspected using light microscope (Fish Olympus phase contrast microscope). For microscopic spore characterization, slide culture technique and phenol staining were used.

### Molecular identification

#### DNA extraction

The genomic DNA of the isolates was extracted from four days of old mycelial culture grown on PDA following a quick and safe fungal DNA extraction method [28] in the laboratory of molecular biology, University of Chile, Santiago. Approximately 400 mg of mycelia grown on the PDA were transferred to a 1.5-ml Eppendorf tube containing 0.5 ml of DNA extraction buffer (1 M KCl; 100 mM Tris-HCl; 10 mM EDTA) using sterile toothpick. Soon after mycelia transfer, mycelial tissue was pulverized by using sterile plastic pestle fitted with an instrument Black and Decker portable electronic drill (American manufacturer of power tools, Stanley Black & Decker, Inc.) for 2 to 3 s. The lysates were centrifuged at 12,000 *g* for 10 min in order to separate cell debris and contaminants from the supernatant. The DNA-containing supernatant was carefully transferred to another 1.5-ml Eppendorf tube containing 0.3 ml of 2-propanol and mixed through tube inverting and centrifuged at 13,000 *g* for 10 min. After discarding supernatant, pellet in the Eppendorf tube was gently washed with 0.7 ml of ethanol (70%) and allowed for ethanol evaporation at room temperature for 15 min. The pellet of DNA was dissolved by 100 μl of 20 mM Tris solution through gentle tapping. The amount of DNA in the suspension was quantified by transferring 2 μl aliquots on to nanodrop microplate in duplicate using an instrument, BioTek Synergy_2_^TM^ Multi-mode Microplate Reader, controlled by Gen5^TM^ Data analysis software, USA. Furthermore, the purity of DNA was checked by running the PCR products under agarose gel electrophoresis and stored at − 20 °C for further activities.

#### PCR amplification

The polymerase chain reaction (PCR) of DNA was performed by using ITS1 and ITS4 primers. ITS1 (TCCGTAGGTGAACCTGCGG forward) and ITS4 (TCCTCCGCTTATTGATATGC reverse) were used to amplify the target regions [29]. The master mix was prepared from the components namely 6.8 μl of water, 4 μl of buffer, 1 μl (2.5 mM·μl^-1^) MgCl_2_, 1 μl (0.5 mM·μl^-1^) of dNTP, 0.2 μl (1 U·μl^-1^) of GoTaq polymerase, 2.5 μl (2.5 μM·μl^-1^) of each ITS1 (forward) and ITS4 (reverse) primers, and 1 μl (30 μg μl^-1^) of genomic DNA. The PCR reaction was conducted in a total volume of 20 μl. The thermo cycler settings were adjusted as 4-min initial denaturation at 94 °C followed by 35 cycles of 1-min denaturation at 94 °C, 1-min annealing at 56 °C, and 1-min extension at 72 °C with final extension for 5 min at 72 °C and storage temperature of 4 °C. The product was assayed by electrophoresis on a 2.5% agarose gel with TBE buffer (Tris; Borate; EDTA) at 100 V for 55 min. Then the gel was stained by shaking within 200 ml of TBE buffer supplemented with 10 μl (v/v) of noncarcinogenic dye “SafeView^TM^ Plus” for 50 min and photographed under UV light using eight-mega pixels canon pc1201 digital camera. The PCR products amplified at the volume of 50 μl was purified by using NucleoSpin® Gel and PCR Clean-up kits (Germany), checked for DNA purity using 2.5% of agarose gel electrophoresis and sent to Macrogen Inc. Seoul, South Korea, for sequencing.

Sequences were aligned using CLUSTALx program, edited by using Bioedit software, and the relationship of the isolates with other relatives were checked by sequence using the BLAST search method from NCBI database. The phylogenetic tree was constructed using MEGA4 program, the neighbor-joining method, the maximum composite likelihood model and 1000 bootstrap runs.

#### Screening of isolates

##### Spore germination potential of isolates

The conidial viability of the isolates were checked through germination test using procedures described by Habtegebriel et al. [30]. Spores were collected from the 3 weeks of old culture and transferred into a Falcon tube containing 10 ml of sterile distilled water supplemented with Tween 80 (0.1% v/v). A 100-μl spore suspension that adjusted to 1 × 10^6^ conidia·ml^-1^ using an improved Neubauer hemocytometer was overspread on the fresh PDA and incubated at 25 °C for 24 h. Over germination of the spores on the medium was halted by dispensing 70% of ethanol. From which, 100 spores (both germinated and non-germinated ones) were counted using 40× magnifying light microscope and the experiment were repeated three times.

##### Spore production potential of isolates

The sporulation rates of the isolates on the medium were tested through the plate culture method by incubating at 28 °C under complete dark condition. The plate cultures grown on PDA were checked daily for sporulation initiation since 4 days after initial incubation. Sporulation initiation of each isolate was recorded for 20 consecutive days (day 20 is considered as experimental lasting date), and the experiment was undertaken in triplicates. To select fast-sporulating isolates, the relative sporulation rate (RSR) was calculated using the formula: $\mathrm{RSR}=\frac{\mathrm{Experimental}\ \mathrm{lasting}\ \mathrm{date}}{\mathrm{PCS}\ \mathrm{date}\ \mathrm{of}\ \mathrm{isolate}},$ where PCS (plate culture sporulation) date is the first date that the isolates were started to produce the spore on the PDA medium. Experimental lasting date is the final date of the experiment schedule, which is just at the 20th day of first inoculation.

##### Pathogenicity screening of isolates using *Galleria mellonella*

Pathogenic isolates were screened using third instar larvae of *G. mellonella* [30]. Spores from 3 weeks grown culture were collected and adjusted to conidial concentration of 1 × 10^7^ ml^-1^ and suspended into Falcon tubes containing 10 ml of distilled water plus Tween 80 (0.1% v/v). Twenty of third instar larvae were dipped into spore suspensions for 15 s, air dried for 10 min under laminar airflow hood, and transferred into sterile small jars filled with a mixture of wheat bran (25 g), honey (40 g), and glycerol (90 ml), separately.

Inoculated jars with larvae were incubated at room temperature for 10 days under dark condition, from which dead larvae were collected every 3 days, surface sterilized, and transferred into sterilized plates lined with moistened tissue paper. Moisture content of the tissue paper was adjusted using sterile water spray to enhance mycelial outgrowth over the larval cadavers. Other twenty larvae were also dipped in sterilized water plus Tween 80 (0.1% v/v) and incubated at the same condition as a control, and the experiments were done in triplicates. Based on these screening procedures, the cumulative biological efficacy index (BEI) was computed by using formula stated by Sain et al*.* [31] with some modification; BEI (%) = 37 (SG) + 13 (RSR) + 50 ( LM), where SG means spore germination, RSR means relative sporulation rate, and LM means larval mortality of *G. mellonella* in 10 days post inoculation of spores.

#### Pathogencity bioassay of isolates against *Tuta absoluta*

Twelve of isolates, which were screened based on BEI values (isolates indicated by * in Table 1), were used for pathogenicity evaluation against second and third instar larvae of *T. absoluta* [32]. These isolates were grown on the PDA for 20 days, and their spore suspension were adjusted to 1 × 10^7^ conidia·ml^-1^ consecration using sterile water plus Tween 80 (0.1% v/v) as before. The tomato leaves were surface sterilized using ethanol (70%) for 3 min and rinsed three times with sterile distilled water. The tomato leaf petioles were tied using UV-sterilized cotton wool to retain water and prevent leaf drying. The leaves were kept in sterile plastic plates (12 cm in diameter) and sprayed with 3 ml of the 1 × 10^7^ spores·ml^-1^ concentration and air dried under laminar airflow hood for 3 min. Then twenty of each second and third instar larvae of *T. absoluta* were released over the spore-sprayed leaves, and the same number of larvae were released over surface-sterilized leaves that sprayed with Tween 80 (0.1% v/v) plus water as a control. All plates were incubated at room temperature for 7 days under dark condition. Mortality of larvae was checked daily, and dead larvae were surface-sterilized and transferred into other sterile plastic plates containing moistened tissue paper to see mycosis for 20 days and experiment was repeated three times. The data for larval mortality were calculated by using the Abbettos formula [33].

**Table 1:** Percentage of spore germination, culture sporulation in 20 days, larval mortality of *Galleria mellonella* at 1 × 10^7^ spores·ml^-1^ concentration within 10 days, and biological efficacy index of the isolates

Isolates	Genera	Soil	SG (%) ± SE	DPCS ± SE	RSR	LM (%) ± SE	BEI (%) ± SE
AAUB76^*^	*Beauveria*	Cropland	94.0 ± 2.60 ^a^	10.7 ± 2.44^a^	1.876	100 ± 0.00^a^	85.0 ± 1.25^a^
AAUB28^*^	*Beauveria*	Cropland	94.0 ± 1.87^ab^	11.0 ± 1.60^a^	1.818	100 ± 0.00^a^	85.0 ± 0.75^a^
AAUB90^*^	*Beauveria*	Cropland	90.7 ± 3.17^b^	11.2 ± 2.02^ab^	1.792	100 ± 0.00^a^	83.8 ± 1.98^ab^
AAUB19^*^	*Beauveria*	Cropland	93.3 ± 1.43^ab^	11.5 ± 0.39^a^	1.739	100 ± 0.00^a^	84.8 ± 1.67^a^
AAUB39^*^	*Beauveria*	Cropland	92.0 ± 3.23^b^	12.7 ± 2.32^ab^	1.580	100 ± 0.00^a^	84.3 ± 2.12^a^
AAUB45	*Beauveria*	Cropland	81.3 ± 4.12^ef^	14.0 ± 0.86^c^	1.429	90.0 ± 3.23^b^	75.3 ± 3.41^bc^
AAUB46	*Beauveria*	Cropland	90.7 ± 2.28^b^	11.3 ± 2.43^ab^	1.765	90.0 ± 3..32^b^	78.7 ± 2.87^b^
AAUB22	*Beauveria*	Cropland	86.3 ± 2.45^c^	15.0 ± 0.82^d^	1.333	91.3 ± 1.67^b^	77.8 ± 3.21^b^
AAUB49	*Beauveria*	Cropland	92.0 ± 4.00^b^	15.0 ± 1.09^d^	1.333	86.7 ± 1.67^dc^	77.5 ± 2.45^b^
AAUB25	*Beauveria*	Cropland	89.0 ± 2.09^bc^	12.2 ± 3.00^ab^	1.645	86.7 ± 1.78^dc^	76.5 ± 2.06^b^
AAUB70	*Beauveria*	Cropland	87.3 ± 3.18^c^	11.2 ± 2.08^ab^	1.792	89.0 ± 4.02^bc^	77.1 ± 3.56^b^
AAUB08	*Beauveria*	Cropland	91.0 ± 2.06^b^	14.8 ± 1.45^bc^	1.349	83.3 ± 1.07^d^	75.5 ± 1.12^bc^
AAUB60	*Beauveria*	Cropland	84.3 ± 1.65^d^	12.7 ± 1.12^ab^	1.580	86.7 ± 1.67^de^	74.7 ± 1.89^bc^
AAUB85	*Beauveria*	Cropland	85.7 ± 4.23^cd^	13.0 ± 2.09^b^	1.538	80.0 ± 2.00^de^	71.9 ± 3.80^c^
AAUB18	*Beauveria*	Cropland	85.7 ± 2.68^cd^	13.0 ± 0.34^b^	1.538	76.7 ± 2.78^fg^	70.2 ± 4.56^c^
AAUB05^*^	*Beauveria*	Grassland	94.0 ± 2.09^a^	13.6 ± 0.64^ab^	1.467	100 ± 0.00^a^	85.0 ± 2.34^a^
AAUB03^*^	*Beauveria*	Grassland	96.7 ± 3.06^a^	10.8 ± 1.44^a^	1.847	100 ± 0.00^a^	86.0 ± 1.92^a^
AAUB06^*^	*Beauveria*	Grassland	91.3 ± 1.10^b^	14.3 ± 2.00^c^	1.396	100 ± 0.00^a^	84.0 ± 2.34^ab^
AAUB07	*Beauveria*	Grassland	43.3 ± 3.19^gh^	14.0 ± 1.53^c^	1.429	92.2 ± 4.08^b^	62.3 ± 5.03^d^
AAUB59^*^	*Beauveria*	Forest	92.3 ± 1.45^ab^	11.2 ± 1.15^ab^	1.792	100 ± 0.00^a^	84.4 ± 2.57^a^
AAUB24^*^	*Beauveria*	Forest	97.0 ± 4.20^a^	12.0 ± 1.10^ab^	1.667	100 ± 0.00^a^	86.1 ± 1.56^a^
AAUB23^*^	*Beauveria*	Forest	88.3 ± 2.45^bc^	14.3 ± 1.27^c^	1.397	100 ± 0.00^a^	82.9 ± 3.67^ab^
AAUB29^*^	*Beauveria*	Forest	91.7 ± 1.89^b^	12.3 ± 0.72^ab^	1.622	96.7 ± 3.67^ab^	82.5 ± 2.56^ab^
AAUB26	*Beauveria*	Forest	91.0 ± 4.29^b^	11.0 ± 2.10^ab^	1.818	90.0 ± 2.70^b^	78.9 ± 3.78^b^
AAUB69	*Beauveria*	Forest	90.7 ± 2.54^b^	16.0 ± 1.78^e^	1.250	86.7 ± 3.78^cd^	77.0 ± 3.86^b^
AAUB09	*Beauveria*	Forest	90.7 ± 3.00^b^	15.0 ± 1.60^d^	1.333	86.7 ± 3.67^cd^	77.1 ± 5.12^b^
AAUB20	*Beauveria*	Forest	94.7 ± 0.89^ab^	14.7 ± 2.02^cd^	1.364	83.3 ± 3.67^cd^	76.9 ± 2.67^bc^

Legends**:** *DPCS*, date of plate culture sporulation; *SG*, spore germination; *LM*, larval mortality; *SE*, standard error; ^*^ selected isolates; *RSR*, relative sporulation rating; *BE*, biological efficacy. The same letters in the columns (a, b, c, d, e, f, g, and h) showed the mean values without significant difference at *p* ≤ 0.05

#### Spore production potential of isolates over larval cadavers of *Tuta absoluta*

Plates with larval cadaver of third instar from the former experiment were incubated at room temperature under dark condition to determine spore concentration after 20 days of incubation. The spore concentration was checked by stirring sporulated larval cadaver within 1 ml of sterile water and Tween 80 (0.1% v/v) mixture from five folds of dilution under 40× magnifying microscope using a hemocytomere, and total spore was determined per volume of initial suspension concentration [34].

#### Dose response of isolates against *Tuta absoluta*

The most pathogenic eight isolates of *Beauveria*, which were selected based on their performance of pathogenicity bioassay (isolates indicated by # in Table 2), were evaluated for concentration (LC_50_ and LC_90_) mortality response and time taken to kill 50% (LT_50_) [35]. The stock spore suspension of each isolate was prepared and vortex mixed in sterile distilled water of Tween 80 (0.1% v/v) and adjusted to concentrations of 1 × 10^4^, 1 × 10^5^, 1 × 10^6^, 1 × 10^7^, and 1 × 10^8^ spores·ml^-1^.

**Table 2:** Pathogenicity percentage of isolates against second and third larval instars of *Tuta absoluta* at 1 × 10^7^ spores·ml^-1^ concentration in 7 days post inoculation and sporulation potential over the cadavers

Screened isolates	Species	Accession number	2nd Instar LM ± SE	3rd Instar LM ± SE	Spore conc. per cadaver
AAUB03^#^	*B. bassiana*	MT588402	94.7 ± 3.08^a^	93.3 ± 2.00^a^	1.6 × 10^7^
AAUB29^#^	*B. bassiana*	MT588415	92.0 ± 1.20^a^	95.0 ± 3.05^a^	1.1 × 10^7^
AAUB28^#^	*B. bassiana*	MT588414	95.7 ± 2.67^a^	95.0 ± 2.12^a^	2.1 × 10^7^
AAUB76^#^	*B. bassiana*	MT588421	88.3 ± 1.09^ab^	85.0 ± 3.10^ab^	1.5 × 10^6^
AAUB24	*B. bassiana*	MT588411	81.7 ± 3.62^c^	84.0 ± 2.36^ab^	8.3 × 10^6^
AAUB05	*B. bassiana*	MT588403	71.0 ± 2.01^de^	75.3 ± 3.76^c^	4.6 × 10^6^
AAUB59^#^	*B. bassiana*	MT588419	85.0 ± 2.50^b^	89.0 ± 2.24^a^	1.2 × 10^7^
AAUB39^#^	*B. bassiana*	MT588416	86.7 ± 4.00^b^	83.3 ± 3.98^b^	1.2 × 10^7^
AAUB46	*B. bassiana*	MT588417	73.0 ± 2.30^d^	71.7 ± 3.61^d^	7.3 × 10^6^
AAUB19^#^	*B. bassiana*	MT588408	94.7 ± 1.08^a^	92.0 ± 4.10^b^	3.5 × 10^7^
AAUB90^#^	*B. bassiana*	MT588422	92.0 ± 1.20^a^	90.0 ± 3.02^ab^	1.2 × 10^7^
AAUB06	*B. bassiana*	MT588404	65.7 ± 2.67^f^	68.3 ± 1.89^d^	5.9 × 10^6^

Legends: *LM*, larval mortality; *SE*, standard error; ^**#**^ reselected isolates for dose determination. The same letters in the columns (a, b, c, d, e, and f) showed the mean values without significant difference at *p* ≤ 0.05

This test was undertaken on third instar larvae of *T. absoluta* following the same procedure as before. Median lethal time (LT_50_) calculation was performed only for three intermediate concentrations (1 × 10^4^, 1 × 10^6^, and 1 × 10^8^ spores·ml^-1^) to see the effects of low, medium, and high spore concentrations on times required to kill 50% of larvae, and all treatments were replicated three times. Median lethal concentration (LC_50_) was analyzed by using the probit analysis software of SPSS version 25, and the concentration responses of each replicates were checked for estimation of lethal time to kill 50% (LT_50_) of exposed larvae.

#### Chitinolytic enzyme production of isolates

Out of isolates evaluated for dose response, five (AAUB03, AAUB19, AAUB28, AAUB78, and AAUB90) isolates that scored relatively short LT_50_ and lowest LC_50_ values were tested for cuticle-degrading enzyme production on plate culture and determined by examination of clear zone formation. The 1% of olive oil for lipase, 0.5% of casein for protease, and 0.5% of colloidal chitin for chitinase productions were added into autoclaved and tempered PDA medium, separately. After trough mixing, the medium was poured into 12-cm diameter of Petri dish and allowed for solidification.

The spores from 20 days of old culture were collected by sterile spatula scraping and suspended into 10 ml of sterile distilled water supplemented with Tween 80 (0.1% v/v) and mixed through vortexing in a Falcon tube. The spore concentrations in the suspensions were adjusted to 1 × 10^7^ conidia·ml^-1^ using improved Neubauer hemocytometer, and 1 ml of each suspension was transferred to Eppindorf tubes containing potato dextrose broth and incubated at 28 °C for 24 h to initiate spore germination. Growth-initiated spores of each isolates were spotted on PDA medium by impregnating UV-sterilized cotton buds (fohnsoni®). Inoculated plates sealed with laboratorial film (Parafilm®) and incubated at 28 °C in complete dark condition. The clear zone formation on the medium was checked, and if clear zone is formed, the isolate is considered as an enzyme producer, and the size of the clear zone directly correlates with the amount of enzyme produced.

#### Effect of temperature on the biology of the isolates

The effect of temperature on spore germination of these abovementioned five effective isolates were further evaluated as before by incubating at 15, 20, 28, 35, and 40 °C in complete dark conditions, whereas radial growth of the fungi was determined by using the mycelium disk plating method. The cork borer (6 mm in diameter)–excised mycelium from 72 h of old plate culture were placed on the center of fresh PDA medium, and incubated at 15, 20, 28, 35, and 40 °C under complete dark condition. The diameter of the growing colony was measured at the 12th day after initial inoculation using a ruler, and plates incubated at 28 °C were used as control. The sporulation rate of isolates on different temperature was determined from the culture plates used for radial growth. The starting date of sporulation on the plate was checked side by side with radial growth, and it is extended up to 25 days of first incubation. The date of the first start of sporulation was taken as sporulation beginning date for that specific isolate.

### Statistical data analysis

Spore germination, sporulation date, screening test, and pathogenicity test results were analyzed by using one way analysis of variance (ANOVA) and SPSS software version 25 statistical programs. Mean separations were calculated using Tukey’s honestly significant difference (HSD) test when the values were significant at 푝 ≤ 0.05.

## Results

### Isolation and identification of *Beauveria* isolates

In this study, 43 soil samples were tested for the presence of the entomopathogenic *Beauveria* species, of which 27 (63%) were positive for the same (Table 1). Most of them (15 isolates) were isolated from cropland soil, followed by forest (8 isolates) and grassland (4 isolates) soils. All of these fungal isolates produced white conidial masses on the plate culture and had smooth plate reverses showing the distinct morphological features of *Beauveria* species. Microscopic spore morphology examination demonstrated that these isolates have circular and small-sized spores (data not shown). Although the cultural and morphological distinctiveness of *Beauveria* clearly gives inference for genus level identification*,* structural similarity and lack of typical phenotypic attributes at species level required genetically supported identification.

**Fig. 1:**
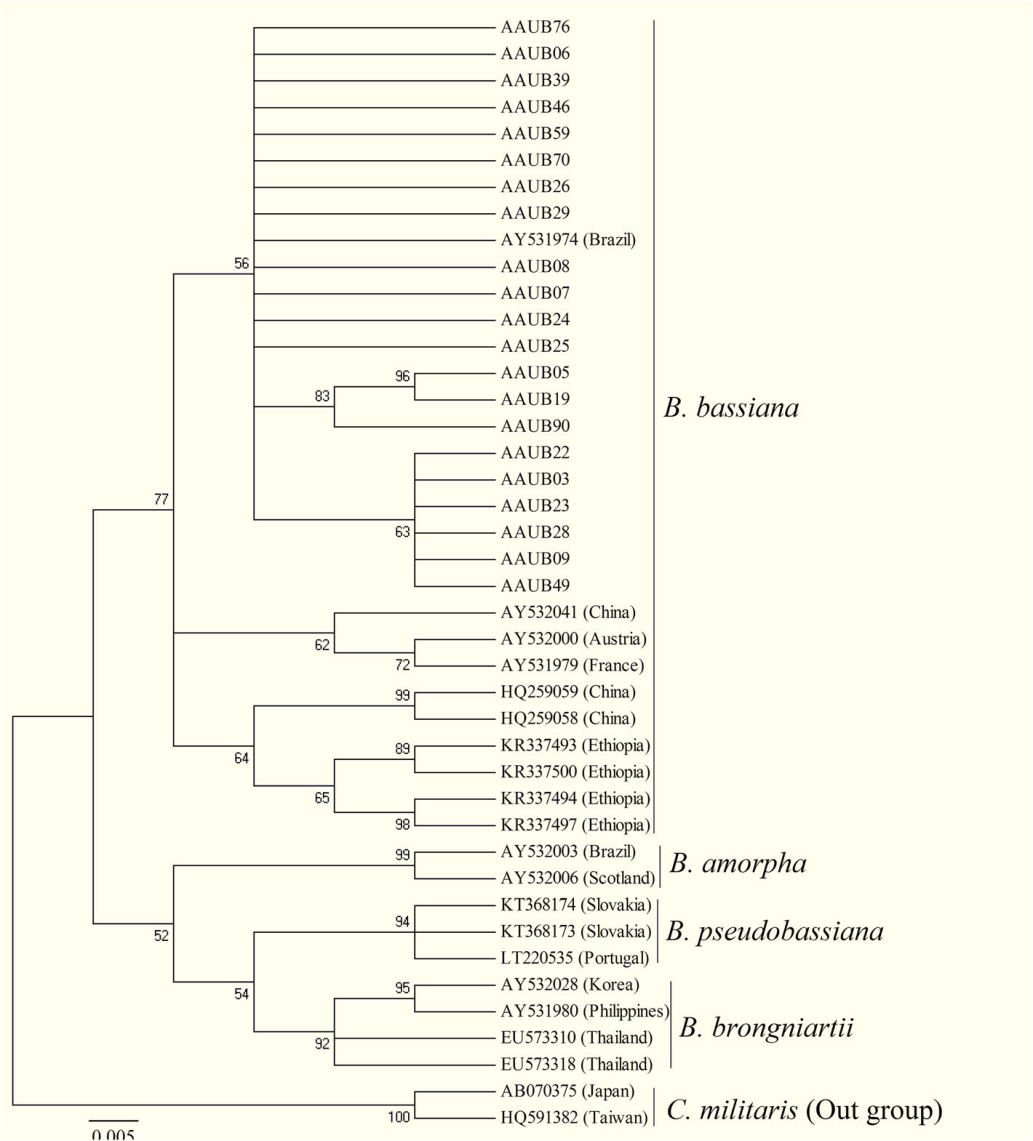
Molecular phylogenic analysis of ITS region of *Beauveria* isolated from Ethiopian soil and other related sequences retrieved from GenBank. Maximum likelihood phylogenetic tree (MEGA4.1) based on ITS sequences from *Beauveria* species using the neighbor-joining model to construct the tree and the Jukes–Cantor sequence evolution algorithm, with 1000 bootstrap replications. Bootstrap values above 50% are shown. The sequences of our isolates were deposited to NCBI database with accession numbers ranging between MT588402 and MT588422, whereas accession numbers of other previously identified strains were presented on the tree followed by countries in parenthesis

Therefore, the ribosomal internal transcribed spacer (ITS1-8.5S-ITS2) rDNA of all isolates were subjected to conventional PCR amplification, and out of these, 21 isolates showed clear and informative band formation with 554 bp (data not shown). The amplified genes of these 21 isolates were sequenced in both direction, and BLAST search results showed all the isolates had 99–100% similarity with previously documented *B. bassiana*. These sequences with other 20 (8 sequences obtained from Rehner and Buckley [36], 8 directly from Genbank, and 4 from Belay et al. [18]) ITS sequences of isolates retrieved from the database showed concordant topology (Fig. 1)*.* The constructed phylogenetic tree showed the isolate category, and our isolates were grouped into three categories. Out of 21 isolates, twelve, three, and six isolates showed 56, 83, and 63% bootstrap support with each other, respectively. Two strains under the second category (AAUB05 and AAUB19) showed very distant evolutionary origin as compared with others with strong (96%) support with each other although they were isolated from different (grassland and cropland) sources of soil. Moreover, almost none of the isolates showed relation with previously identified strains, rather, one isolate from Brazil (*B. bassiana-*AY531974) matched our isolates with bootstrap support of 56% (Fig. 1), and therefore, this shows that our isolates were nearly monophyletic with each other and distinctive from the others.

### Screening of potential isolates

Morphologically and molecularly identified isolates of *B. bassiana* were first screened based on their spore viability, sporulation rate, and pathogenicity against their host insect (*G. mellonella*) through the computation of the biological efficiency index (BEI). All isolates displayed more than 80% of spore germination by showing the significant difference with one another at *p* ≤ 0.05, except isolate AAUB07 with a low spore germination of 43% (Table 1). They attained sporulation (DPCS) within 10 to12 days with relative sporulation ratings (RSR) of 1.2 to 1.8 on the culture plate and scored 76.7–100% of mortality on larvae of *G. mellonella*. The computed biological efficiency index (BEI) of isolates was reneged between 62 and 86%, defined on cumulative effect of all the above-working parameters combined (Table 1).

Out of the isolates explored in this study, 12 (44%) isolates scored BEI values of above 80% and screened for further work. These isolates were effective in larval mortality (LM) of 96.7 to 100% against *G. mellonella* in 10 days of post inoculation. From these, five (AAUB28, AAUB03, AAUB24, AAUB05, and AAUB76) isolates scored highest (85 to 86.1%) BEI values, and other seven isolates scored between 82 and 84.8% BEI values as indicated by asterisk (*) in Table 1. These twelve isolates were used for further pathogenicity bioassay against *T. absoluta*.

### Pathogenicity bioassay against *Tuta absoluta*

It is interesting to note that most of the prescreened isolates were pathogenically effective against *T. absoluta* at 1 × 10^7^spores·ml^-1^concentration in 7 days post inoculation (Table 2). Almost all of these isolates rated moderate (65.7%) to high (95.7%) mortality against both second and third instar larvae of *T. absoluta*. Five of the isolates (AAUB03, AAUB28, AAUB29, AAUB19, and AAUB90) were highly effective and scored 95, 95, 93.3, 92, and 94.7% of mortality against either of the instar larvae, respectively. The best isolate, AAUB28, was equally pathogenic against both second and third instar larvae, and scored 95.7 and 95% of mortality, respectively. However, the mean pathogenicity among isolates showed marked statistical difference with *F* (26, 52) = 8.97, *p* < 0.001 for second instar and *F* (14, 25) = 8.123, *p* < 0.001 for third instar larvae. The sporulation potential of these isolates was considerable and produced 4.6 × 10^6^ to 3.5 × 10^7^ spores·ml^-1^ over the larval cadavers (Table 2).

**Fig. 2:**
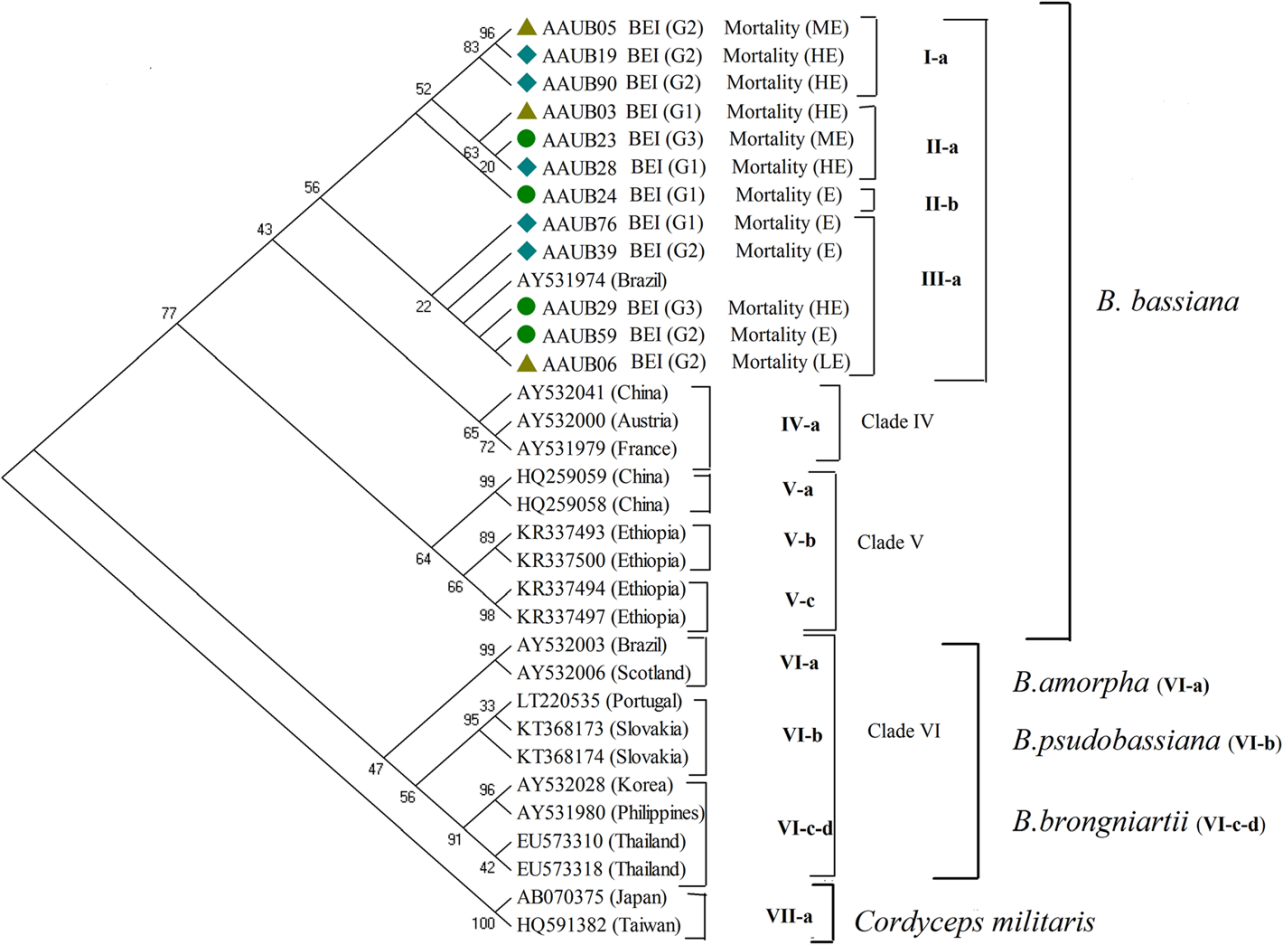
This cladogram showed the characteristics of twelve Ethiopian potent isolates. First sources of isolates, from grassland soil (▲), cropland soil (♦), and forest soil (●) followed by codes for each isolate and biological efficacy index showing that if BEI > 85 = G1, BEI: 83–85 = G2, BEI: 80–83 = G3. Finally, percentage of mortality against *Tuta absoluta* was indicated, such as if value is < 70% (less effective, LE), 70–80% (moderately effective, ME), 80–90% (highly effective, HE)

Furthermore, all of these selected isolates of *B. bassiana* were polyphyletic in source, biological efficiency, and pathogenicity (Fig. 2). For instance, AAUB19 and AAUB90 strains isolated from the cropland soil scored highly effective (HE) mortality against third instar larvae of *T. absoluta*, whereas AAUB05 isolated from the grassland soil scored moderate (ME) lethality, and all are categorized under G2 of BEI. Most G1 strains obtained from three soil types were scored moderate (ME) to high (HE) lethality against *T. absoluta* and distributed in different categories. The remaining five strains were isolated from different types of soil; have BEI of G1, G2, and G3; scored moderate (ME) lethality; and grouped in one category (III-a). In general, none of the isolates showed congruence with respect to sources, BEI, and pathogenicity, rather showed intermixed distribution in the tree (Fig. 2).

### Virulence of isolates against *Tuta absoluta*

In this study, eight of the most pathogenic strains of *B. bassiana* were evaluated to determine the effect of the spore concentration against third instar larvae of *T. absoluta.* These strains scored median lethal time (LT_50_) values ranging between 3.3 and 5.67 days at 10^4^ spores·ml^-1^ and 1.93 to 3.17 days at 10^8^ spores·ml^-1^ concentrations (Table 3). Three strains, AAUB03, AAUB19, and AAUB28 attained the LT_50_ values at the same 3.33 days, whereas strain AAUB76 scored relatively extended (5.76 days) LT_50_ values. Similarly, corresponding LC_50_ values for all selected isolates ranged between 1.5 × 10^3^ and 1.1 × 10^5^ spores·ml^-1^and LC_90_ value of 2.8 × 10^5^ and 1.8 × 10^7^ spores·ml^-1^ (Table 3). The isolates AAUB03, AAUB28, AAUB19, and AAUB90 scored LC_50_ values of 5.3, 1.3, 1.8, and 2.6 × 10^3^, and AAUB29, AAUB76, and AAUB39 scored 6.5, 9.3, and 1.8 × 10^4^ spores·ml^-1^, respectively. The least LC_50_ value (1.1 × 10^5^ spores·ml^-1^) was scored by isolate AAUB59. In general, concurrent larval mortality increase was recorded in all evaluated isolates as spore concentration and exposure time increased.

**Table 3:** The LT_50_ determination and summary of probit analysis to determine LC_50_ and LC_90_ concentrations *of Beauveria bassiana* strains against the third instar of *Tuta absoluta* larvae in 10 days post incubation

	LT_50_ (mean ± SE)							
	AAUB03	AAUB29	AAUB28	AAUB76	AAUB59	AAUB39	AAUB19	AAUB90
Spores·ml^-1^								
10^4^	3.33± 0.58^a^	5.33 ± 0.58^c^	3.33± 0.58^a^	5.67 ± 0.58^cd^	5.00 ± 1.00^c^	3.67 ± 0.58^ab^	3.33 ± 0.58^a^	5.00 ± 1.00^c^
10^6^	2.33± 0.58^a^	2.57 ± 0.51^a^	2.50± 0.00^a^	2.83 ± 0.29^ab^	2.93 ± 0.12^ab^	2.17 ± 0.29^a^	2.33 ± 0.29^a^	4.00 ± 0.00^c^
10^8^	1.93± 0.12^a^	2.00 ± 0.00^a^	2.20± 0.35^a^	2.33 ± 0.29^a^	2.50 ± 0.50^ab^	2.00 ± 0.35^a^	2.00 ± 0.62^a^	3.17 ± 0.29^c^
Summary of probit analysis								
LC_50_	5.3 × 10^3^	6.5 × 10^4^	1.5 × 10^3^	9.3 × 10^4^	1.1 × 10^5^	1.8 × 10^4^	1.8 × 10^3^	2.6 × 10^3^
95% FL	1.3 × 10^3^ –7 × 10^4^	2.2 × 10^4^–1.5 × 10^5^	8.6 × 10^2^–4.9 × 10^3^	9.9 × 10^3^–1.3 × 10^4^	2 × 10^4^–3.3 × 10^5^	3.9 × 10^3^–6 × 10^4^	1 × 10^3^–6.1 × 10^4^	1.2 × 10^3^–3.1 × 10^4^
LC_90_	2.8 × 10^5^	1.7 × 10^6^	2.9 × 10^5^	1.6 × 10^6^	1.8 × 10^7^	1.6 × 10^7^	7.6 × 10^5^	1.2 × 10^7^
95% FL	2.1 × 10^5^ –6.3 × 10^6^	6.3 × 10^5^–1.1 × 10^7^	1.9 × 10^4^–2.9 × 10^6^	1.8 × 10^5^–2.7 × 10^7^	4.2 × 10^6^–2.8 × 10^8^	2.2 × 10^6^–3.1 × 10^8^	7.1 × 10^4^–2.1 × 10^7^	1.5 × 10^6^–7.3 × 10^8^
Int ± SE	1.32 ± 0.91	4.33 ± 0.89	0.85 ± 0.83	1.16 ± 0.72	2.91 ± 0.66	1.54 ± 0.64	0.8 ± 0.75	1.19 ± 0.63
S ± SE	0.49 ± 0.18	0.9 ± 0.17	0.39 ± 0.16	0.39 ± 0.13	0.58 ± 0.11	0.39 ± 0.11	0.36 ± 0.14	0.35 ± 0.11
*p* value	< 0.001	< 0.001	< 0.012	< 0.003	< 0.001	< 0.001	< 0.001	< 0.001

Legends: *FL*, fiducial limit; *SE*, standard error; *LT*, lethal time taken to kill fifty of experimental organisms; *LC*, lethal concentration; *Int*, intercept; *S*, slope. The same letters in the columns (a, b, c, d, e, and f) showed the mean values without significant difference at *p* ≤ 0.05

### Chitinolytic enzyme production of effective isolates

Out of eight potent isolates, five isolates that scored short LT_50_ and lowest LC_50_ values were evaluated about their pathogenicity-enhancing chitinolytic enzyme production potential using the clear zone determination method. The strains of *B. bassiana* which are capable to produce lipase, protease, and chitinase enzymes were highly pathogenic against their target pests. Therefore, five of our isolates were lipase, protease, and chitinase positive and showed clear zone formation on respective medium for each enzyme. However, the clear zone formation for each enzyme type varied among isolates (Table 4). Two isolates (AAUB03 and AAUB28) showed large (+++) clear zones on the PDA medium supplemented with casein as compared with other three isolates (Table 4). The large clear zone formation phenomenon may correlate with the amount of protease secreted by the fungal isolates. The remaining three (AAUB29, AAUB19, and AAUB90) isolates that scored 95, 92, and 90% of mortality against third instar larvae of *T. absoluta* formed intermediate (++) clear zones by protease (Table 4). The isolate AAUB03 continued production of better (++) clear zones on the medium supplemented with colloidal chitin than the others, those formed small (+) clear zones. Furthermore, isolates showed very small (+) clear zone formation on the medium supplemented with olive oil that correlates with less production of lipase enzyme (Table 4).

**Table 4:** Extracellular enzyme production of five selected strains on agar culture medium

Isolates	Accession	Lipase	Protease	Chitinase
AAUB03	MT588402	+	+++	++
AAUB28	MT588414	+	+++	+
AAUB29	MT588415	+	++	+
AAUB19	MT588408	+	++	+
AAUB90	MT588422	+	++	+

### Effect of temperature on the biology of isolates

**Fig. 3:**
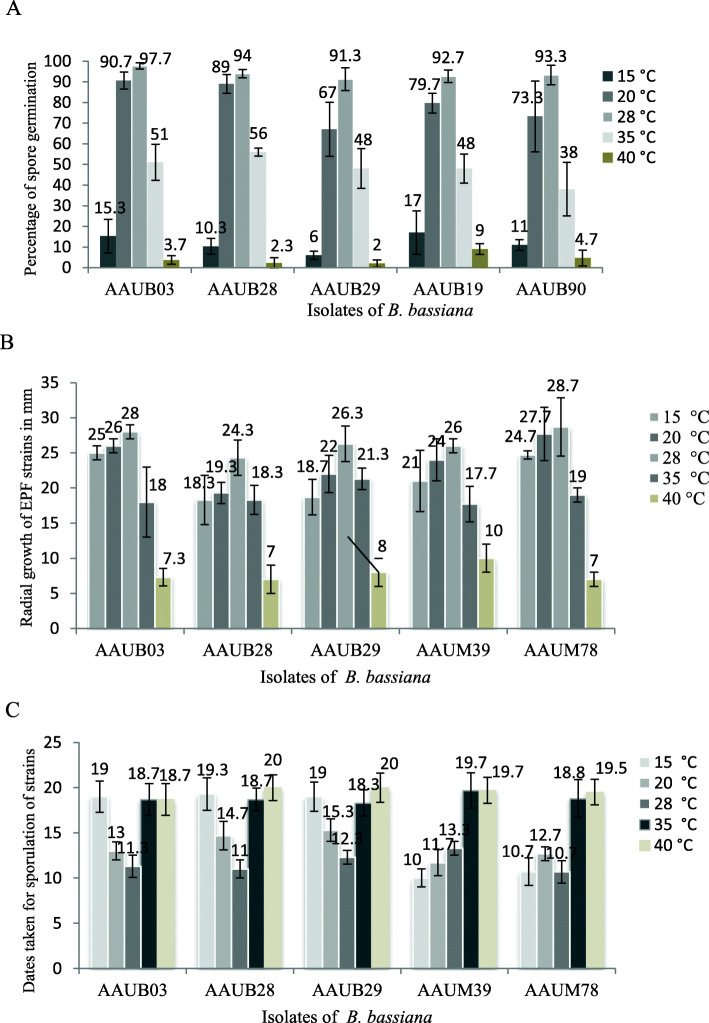
Effect of temperature on the spore germination (**A**) radial growth (**B**), and dates taken for sporulation (**C**) of *B. bassiana* isolates

Temperature is the most important abiotic factor that influences the biology of EPF strains. Isolates that adapt temperatures ranging out of the optimum may be considered to be used in adverse conditions for pest control. Therefore, we have evaluated five of effective isolates for their spore germination, radial growth, and sporulation potential at different temperature classes (Fig. 3). Consequently, all tested isolates showed an effect on conidial germinations between 6 and 17%, and two of them (AAUB03 and AAUB90) showed better (15.3 and 17%) germination at 15 °C (Fig. 3A). All of the isolates showed almost better conidial germination ranging between 67 and 90.7% at 20 °C and above 90% of germination at 28 °C (Fig. 3A). The conidial germination of all the strains was highly affected at 35 °C that reduced almost between 38 and 55.3% germination, and almost none of the spores germinated at 40 °C temperature (Fig. 3A).

Similarly, isolates showed normal radial growth at 20 and 30 °C; however, it was highly affected at 35 °C and 40 °C (Fig. 3B), while the radial growth of isolate AAUB28 was highly affected than the other isolates in all temperature ranges. Interestingly, AAUB03, AAUB19, and AAUB90 showed almost the same radial growth rate with positive control at 15 °C (Fig. 3B). In the same way, low and high temperature range, also affected the sporulation rates of the strains; however, temperature at 20 °C is better tolerated as compared with others (Fig. 3C).

## Discussion

The first stage of infection of insects by entomopathogenic fungi (EPF) is the adhesion of the spore or conidia on the cuticle of the insect. The subsequent germination and generation of a macromolecular structure called appresorium, or the formation of a spore germ tube, induces the secretion of hydrolytic enzymes to begin the degradation of the insect’s integument [37–40]. Since the formation of appressorium in all *B. bassiana* strains is not yet clear [41], some strains of this fungus in contact with artificial surfaces generated an appressorial-like structure under laboratory conditions [42]. Literatures showed that the adherence of *B. bassiana* to the insect cuticle starts the secretion of hydrolytic enzymes, such as chitinase, protease, lipases, and glycosidase that play a crucial role on cuticle degradation [37, 38, 43]. Among these enzymes, the activity levels of chitinase, protease, and lipase have been shown to correlate with the degree of virulence of isolates [44–46].

As mentioned above, the virulence of EPF might be determined by the genetic makeup of the strains based on their adaptation of different geographical and ecological exposures to regulate expression of these hydrolytic enzymes [39, 40, 47]. Therefore, we have checked the production of lipase, protease, and chitinase enzymes by isolates of *B. bassiana* on the medium supplemented with respective substrates; however, the size of the clear zones formed for each types of enzymes vary from isolate to isolate. The variation of extracellular enzyme production within different EPF isolates was common phenomenon [45, 48]. This variation may be due to substrate quality, genetic variation, or adaptive attributes of enzyme-coding genes in the fungal isolates.

Literatures showed that lipase is an important lipolytic enzyme of EPF that facilitates the spore attachment on the integument of insect and starter of hydrolysis of the uppermost component, fatty acid of the cuticle [49–52]. Thus, our isolates formed small clamps around the colony on the medium supplemented with olive oil, showing that isolates were lipase positive, but the production is very low. On the other hand, protease has been identified as an important virulence factor of EPF isolates and is involved in the cleaving of peptide bonds of cuticle proteins and enhances the virulence against targeted insects [53]. Concordantly, the most potent isolates that scored 94 and 95% of mortality against *T. absoluta* showed large clear zone formation, and this implies that the amount of protease might be directly correlated with virulence of isolates. Therefore, their virulence could be associated with their ability of extracellular enzyme production. Other researchers reported high virulence of *B. bassiana* with their ability of producing large amounts of protease [45]. Furthermore, isolates were chitinase positive and formed clear zones on the medium supplemented with colloidal chitin; however, the clear zone formation is varying from isolate to isolate. This enzyme degrades chitin that is the main component of insect cuticle and finalizes the integument degradation following protease activity [54].

Thus, we have screened putative strains of *B. bassiana* from the different sources of local environment to obtain effective candidates for pest control. In this study, 27 isolates of *B. bassiana* were recovered from the cropland soil (55.6%), forest soil (29.6%), and grassland (14.8%) soil (Table 1), which is comparable with other reports on *B. Bassiana* (52.1%) from the soils of eastern Ethiopia, 80% of isolates from the natural forest, and 23% from cropland soils of Jimma Zone [18].

These isolates were analyzed for their genetic relations, and the resulted phylogenetic tree grouped isolates into three distinctive categories in which all are nearly monophyletic as similar to reports of others showing that the variations of most strains of *B. bassiana* are insignificant regardless of geographical locations [36, 55–57]. However, in the present study, isolates of *B. bassiana* showed different categorization and would correspond to new isolates, which is different from previously published Ethiopian isolates [18] because all the isolates previously described in Ethiopia appear in other clades (Fig. 1). This could give an important clue to investigate the most distinctive strains of *B. bassiana* from Ethiopian soil samples. Although isolates are unique from the other previously identified strains, all isolates showed less genetic variation among each other. This might lead to hypothesize that the soil type and sample location may not influence the genetic makeup of the isolates recovered.

In addition, biological characteristics such as spore viability, sporulation rate, and pathogenicity against their host insect are the vital attributes to screen effective strains of entomopathogens [31]. Therefore, we have screened our isolates based on the biological efficiency index determination (31), and 12 (44.4%) of the isolates were scored above 80% of BEI. These isolates achieved more than 90% of spore germination, 90–100% of larval mortality against *G. mellonella*, and sporulated within 10 to 12 days of culturing on the medium with relative sporulation rating (RSR) of 1.3 to 1.8. Similarly, different researchers screened effective EPFs for an agriculturally important pest control using these attributes [58, 59].

All of the screened isolates showed promising (65.7–95.7% and 68.3–95%) mortality rates against second and third instar larvae of *T. absoluta*, respectively. Interestingly, five (AAUB03, AAUB28, AAUB29, AAUB19, and AAUB90) of the isolates were scored 90–95.7% of mortality at 1 × 10^7^ spores·ml^-1^ concentration. This is concurrent with other reports on *B. bassiana* that scored 90% of mortality against *T. absoluta* in Egypt [60]. In addition, recently explored Ethiopian isolate of *B. bassiana* displayed 95.8% of mortality against third instar larvae of *T. absoluta* at 2.5 × 10^9^ spores·ml^-1^ [61]. In all cases, Ethiopian isolates of *B. bassiana* performed better than the commercially formulated products (Beauvitech® WP) that scored 60.8% of larval mortality against *T. absoluta* at 10^8^ spores·ml^-1^ in Rwanda [62]. This could infer that locally screened isolates might be pathogenically effective than commercialized ones, and geographic origin of isolates may determine their pathogenicity.

Furthermore, these pathogenically valued isolates of *B. bassiana* showed almost equal efficacy against both second and third instar larvae of *T. absoluta* by scoring 95.7% and 95% of mortality, respectively, which is contradicting to reports of Tsownara and Port [63] which showed that third instar larvae of *T. absoluta* is more susceptible to *B. bassiana* than the second instar larvae. In addition, these isolates produced considerable (4.6 × 10^6^ to 3.5 × 10^7^ spores·ml^-1^) amount of spores over the dead larval cadavers. This makes that fungal spores are easily disseminated between insects through the process of auto-dissemination. Thus, high sporulation potential of EPF over the cadavers enhanced horizontal pest infection through the self-spore contamination process [64]. Besides, our isolates scored shortest (1.93 to 3.17 days) median lethal time (LT_50_) at 10^8^ spores·ml^-1^, which were different from the two commercially formulated *Beauveria* (Beauvitech® WP and Botanigard® ES) that scored somewhat extended LT_50_ values of 5.2 and 6.6 days at the same spore concentration against *T. absoluta*, respectively [62]. This is at least twice the LT_50_ value of the Ethiopian isolates presented here. However, a former Ethiopian isolate of *B. bassiana* scored a LT_50_ value of 5.2 days at the spore concentration of 2.5 × 10^9^ spores·ml^-1^ [61], which takes considerably longer time to kill 50% of third instar larvae of *T. absoluta* as compared with their spore concentration.

It is interesting to mention that effective isolates of *B. bassiana* in this study scored promising LC_50_ and LC_90_ (1.5 × 10^3^ and 2.8 × 10^5^ spores·ml^-1^) values against third instar larvae of *T. absoluta,* respectively. Similarly, other isolates of *B. bassiana* scored LC_50_ values of 4.5 × 10^5^ and 3.6 × 10^5^ spores·ml^-1^against second and third instar larvae of *T. absoluta*, respectively [65]. Usually, when the spore concentration and exposure time increases concurrently, larval mortality also increases in all evaluated strains of *B. bassiana*, which is comparable with other findings [61, 63, 65].

Although locally isolated EPF strains showed better performance on biocontrol activity, different abiotic factors might influence survival of the bioagents. Temperature is one of the abiotic factors that could influence spore germination, mycelial growth, and spore production of the fungal isolates [66]. We have checked both lower and upper temperature ranges against spore germination, radial growth, and sporulation rate of the some selected potent isolates and temperatures at 20 to 30 °C showed nil effect on spore germination, mycelial growth, and sporulation. Similarly, others reported that temperature ranging at 24–30 °C was optimum for spore germination, mycelial growth, and speculation [54]. However, temperature lower than 20 °C and above 30 °C was highly adverse for spore germination of *B. bassiana* [67], which is concurrent to our study.

## Conclusion

This study explains the presence of more indigenous strains of *B. bassiana* in different soil types of Ethiopia with better entomopathogenic characteristics and that is crucial to develop ecofriendly biopesticides for sustainable agriculture. Molecular analysis of these isolates showed monophyletic attributes about genetic relationships and polyphyletic characters about sources, biological efficacy, and pathogenicity against the host insect. Among the isolates of *B. bassiana*, two (AAUB03 and AAUB28) isolates showed strong efficacy against *T. absoluta* with short LT_50_ and low LC_50_ values. These isolates were chitinolytic enzyme producers and better growers at optimum temperatures. Therefore, these indigenous *B. bassiana* were effective against *T. absoluta* under laboratory conditions; however, further investigation at the actual field is required for mass production.

## Data Availability

The datasets used for this work are available with the corresponding author when legal request is presented.

## Abbreviations

AAU: Addis Ababa University
ANOVA: Analysis of variance
APPRC: Ambo Plant Protection Research Center
BEI: Biological Efficiency Index
BLAST: Basic Local Alignment Search Tool
DPCS: Date of plate culture sporulation
EDTA: Ethylenediaminetetraacetic acid
EPF: Entomopathogenic fungi
HE: Highly effective
IPM: Integrated pest management
ITS: Internal transcribed spacer
JC: Jukes–Cantor
LC: Lethal concentration
LE: Less effective
LM: Larval mortality
LT: Lethal time
NCBI: National Center for Biotechnology Information
NJ: Neighbor-joining
ME: Moderately effective
MEGA: Molecular Evolutionary Genetic Analysis
PCS: Plate culture sporulation
PCR: Polymerase chain reaction
PDA: Potato dextrose agar
RSR: Relative sporulation rate
SE: Standard error
SG: Spore germination
SPSS: Statistical Package for the Social Sciences
TBE: Tris-borate-EDTA
USEPA: United States of Environmental Protection Agency
